# Mucous Fistula and End Colostomy for Emergency Management of Septic Large Bowel Obstruction in a Patient With Rectal Cancer: A Case Report

**DOI:** 10.7759/cureus.106110

**Published:** 2026-03-30

**Authors:** Emily Reinoso, Sarah Villamil, Kian Memari, Nuria Lawson

**Affiliations:** 1 Medicine, Nova Southeastern University Dr. Kiran C. Patel College of Osteopathic Medicine, Fort Lauderdale, USA; 2 Family Medicine, Palmetto General Hospital, Miami, USA; 3 General Surgery, Palmetto General Hospital, Miami, USA

**Keywords:** end colostomy, large bowel obstruction, mucous fistula, rectal cancer, sepsis

## Abstract

Obstructing rectal cancer complicated by sepsis represents a surgical emergency. When definitive oncologic resection is not feasible, alternative diversion strategies are required to safely decompress the bowel and control sepsis. We report the case of a 67-year-old woman with known rectal adenocarcinoma who presented in septic shock due to distal large bowel obstruction. Intraoperative evaluation revealed a completely obstructing fungating rectal mass that precluded Hartmann’s procedure or diverting loop colostomy. Emergent diversion with an end colostomy and concomitant mucous fistula was performed to decompress the excluded distal bowel segment and prevent mucofecal accumulation and perforation. This case highlights mucous fistula creation as a viable and underutilized adjunct in the emergency management of obstructing rectal cancer when standard diversion or resection is not appropriate. Early shared decision-making and timely definitive management of rectal cancer may reduce the risk of high-acuity emergent presentations.

## Introduction

Rectal cancer may present with rectal bleeding, altered bowel habits, or progressive obstruction, and diagnosis requires histologic confirmation [1]. Staging typically includes a thorough clinical history, carcinoembryonic antigen (CEA) measurement, cross-sectional imaging of the chest, abdomen, and pelvis, pelvic magnetic resonance imaging (MRI) with rectal cancer protocol, and endoscopic evaluation. Treatment is guided by tumor stage, anatomic considerations, and molecular features [2].

For early-stage disease, radical resection remains the standard of care. Locally advanced rectal cancers without distant metastasis are commonly managed with total neoadjuvant therapy, consisting of chemotherapy and radiation, or immunotherapy in select molecular subtypes, followed by surgical resection when indicated [1]. Abdominoperineal resection (APR) is required for distal rectal tumors not amenable to sphincter-preserving surgery and results in a permanent end colostomy [3].

When rectal cancer progresses to cause acute large bowel obstruction with systemic infection, emergent surgical intervention is required. In such settings, standard oncologic resection may be unsafe or technically infeasible. We present a case of obstructing rectal cancer complicated by sepsis in which emergent diversion with end colostomy and mucous fistula formation was necessary to safely manage the excluded distal bowel. This case underscores the role of mucous fistula creation as a pragmatic surgical option in select emergency scenarios.

## Case presentation

A 67-year-old woman with a known diagnosis of locally advanced rectal adenocarcinoma presented with acute diffuse abdominal pain, vomiting, and diarrhea. Her medical history was significant for hypertension and type 2 diabetes mellitus. Three months prior, she had been evaluated for definitive surgical management. Preoperative staging had demonstrated tumor extension involving adjacent pelvic structures, including suspected uterine involvement. She had been advised to undergo en bloc APR with hysterectomy. However, after multidisciplinary discussion, the patient elected to undergo hysterectomy alone at an outside institution as part of an initial step in management, deferring the APR at that time with plans for further oncologic treatment. She continued systemic therapy with capecitabine 500 mg orally twice daily.

On presentation, she was hypotensive with clinical features consistent with septic shock. Laboratory evaluation revealed leukocytosis (13.9 × 10³/µL), severe hyperglycemia (502 mg/dL), hyperlactatemia (6.4 mmol/L), and elevated alkaline phosphatase (203 U/L) (Table 1). Initial vital signs included a temperature of 97.9°F, a heart rate of 81 beats/min, and a blood pressure of 94/51 mmHg.

**Table 1:** Initial lab values

Test (unit)	Observed value	Normal range
White blood cell count (×10³/µL)	13.9 × 10³/µL	4.0-11.0 × 10³/µL
Serum glucose (mg/dL)	502 mg/dL	70-99 mg/dL
Lactic acid (mmol/L)	6.4 mmol/L	0.5-2.2 mmol/L
Alkaline phosphatase (U/L)	203 U/L	44-147 U/L

**Figure 1:**
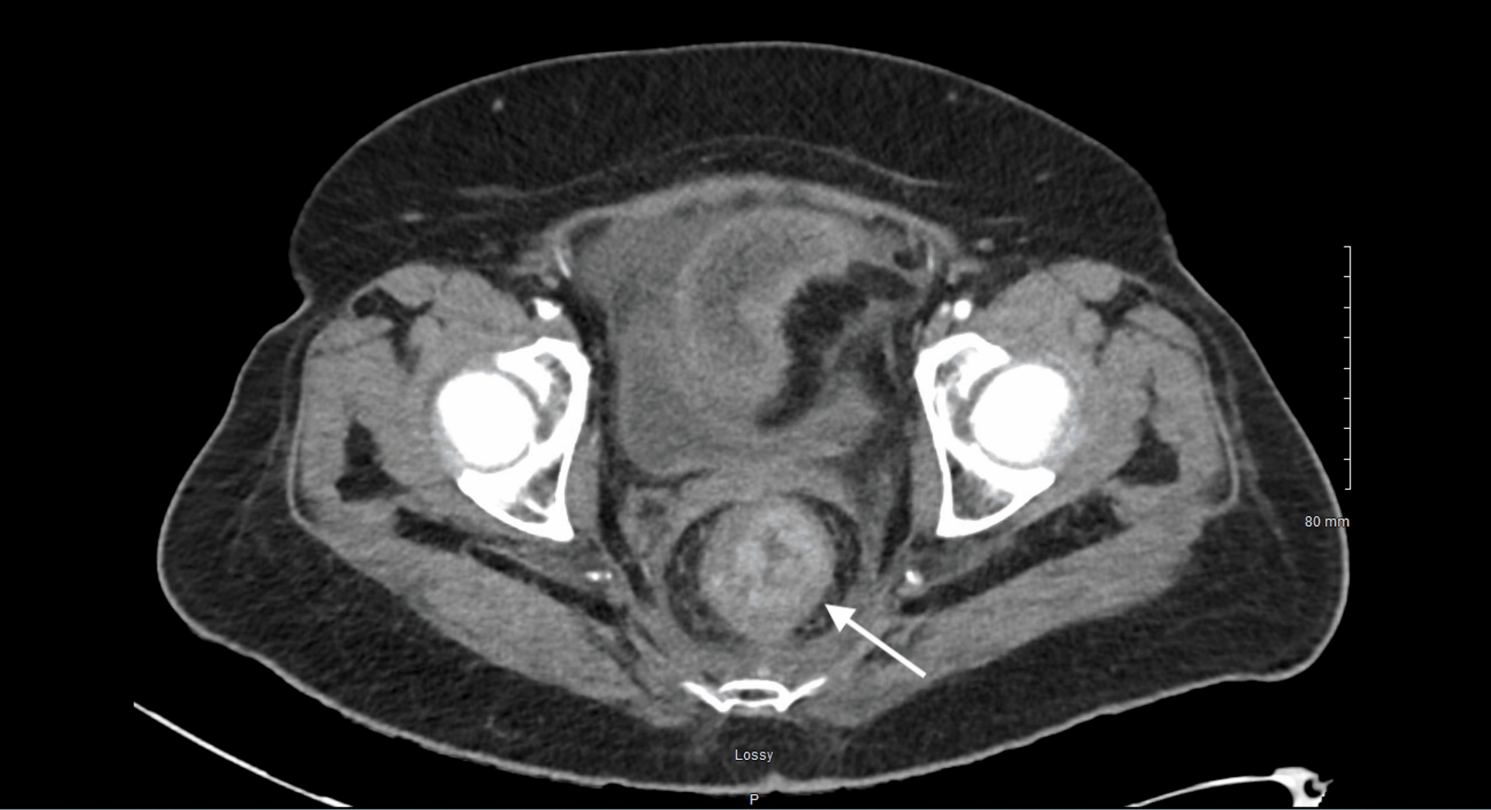
Rectal narrowing The white arrow is pointing to an area of soft tissue fullness within the rectum and anus, which caused significant luminal narrowing.

**Figure 2:**
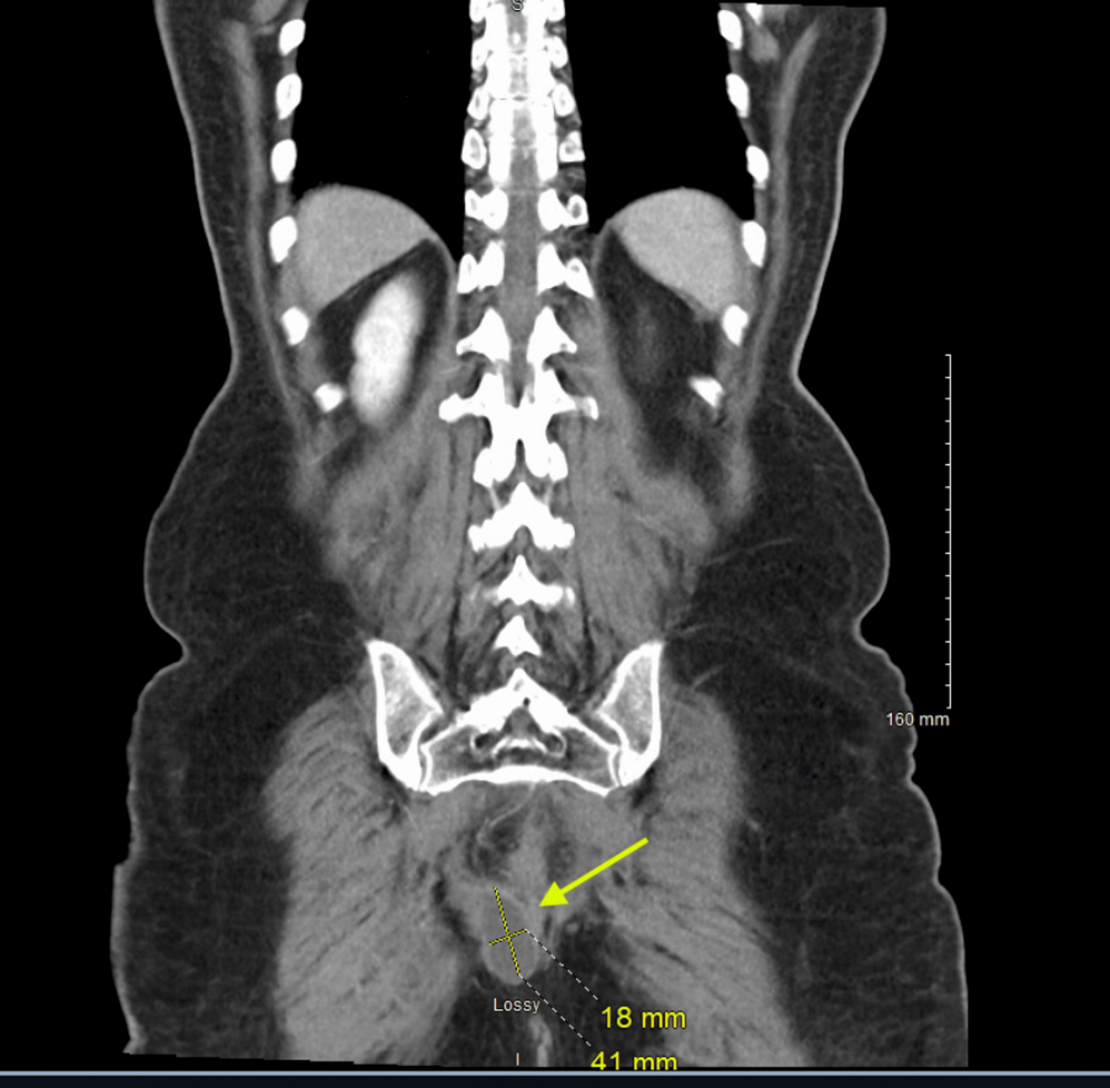
Right perirectal fluid collection The yellow arrow is pointing to a right perirectal fluid collection measuring 39 × 41 × 18 mm.

Physical examination demonstrated diffuse abdominal tenderness with voluntary guarding and hypoactive bowel sounds, without rebound tenderness. A digital rectal examination was performed preoperatively and revealed a firm, obstructing mass in the distal rectum. Contrast-enhanced computed tomography of the abdomen and pelvis demonstrated a distal rectal mass causing significant luminal narrowing consistent with large bowel obstruction, along with a right perirectal fluid collection measuring 39 × 41 × 18 mm (Figures 1 and 2). The imaging findings were consistent with obstructing rectal malignancy rather than an isolated fluid collection.

The patient was admitted to the intensive care unit and managed with aggressive fluid resuscitation, vasopressor support, and broad-spectrum antibiotics. Although procalcitonin testing was not required for diagnosis, the markedly elevated lactate and hemodynamic instability were sufficient to establish septic shock requiring urgent surgical intervention. Despite aggressive intravenous fluid administration, she remained hypotensive and was diagnosed with septic shock, requiring vasopressor support and placement of arterial and central venous lines. Nasogastric decompression was initiated, and the patient was kept nil per os.

Once hemodynamically stabilized, the patient consented to emergent exploratory laparotomy with diverting colostomy and additional procedures as indicated. Intraoperatively, digital and perirectal examination did not demonstrate a drainable abscess; the previously identified fluid collection was attributed to her recent hysterectomy. Rigid sigmoidoscopy confirmed a completely obstructing fungating tumor located approximately 8 cm from the anal verge, consistent with a mid-to-distal rectal lesion. The rectal tumor was found to be densely fixed to the pelvic sidewalls and sacrum, with significant local inflammation and tissue friability. These findings rendered oncologic resection unsafe due to the risk of catastrophic hemorrhage and inability to achieve clear margins. Therefore, the exploratory laparotomy required extensive lysis of adhesions. Redundant loops of sigmoid colon were densely adherent to the posterior peritoneum and pelvic structures adjacent to the rectal mass. Therefore, extensive lysis of adhesions was pursued, and the redundant loops of sigmoid colon were carefully mobilized. Hartmann’s procedure was not feasible because closure of the distal rectal stump would have risked blowout in the setting of distal obstruction and sepsis. A diverting loop colostomy was also not possible due to a lack of bowel mobility and tension on the distal segment. An end colostomy was therefore created at the descending-sigmoid junction in the left lower quadrant. The distal segment was exteriorized as a mucous fistula at the inferior aspect of the laparotomy incision, where sufficient mobility allowed safe exteriorization without tension. This location was selected to minimize additional dissection and operative time in a hemodynamically fragile patient.

**Figure 3:**
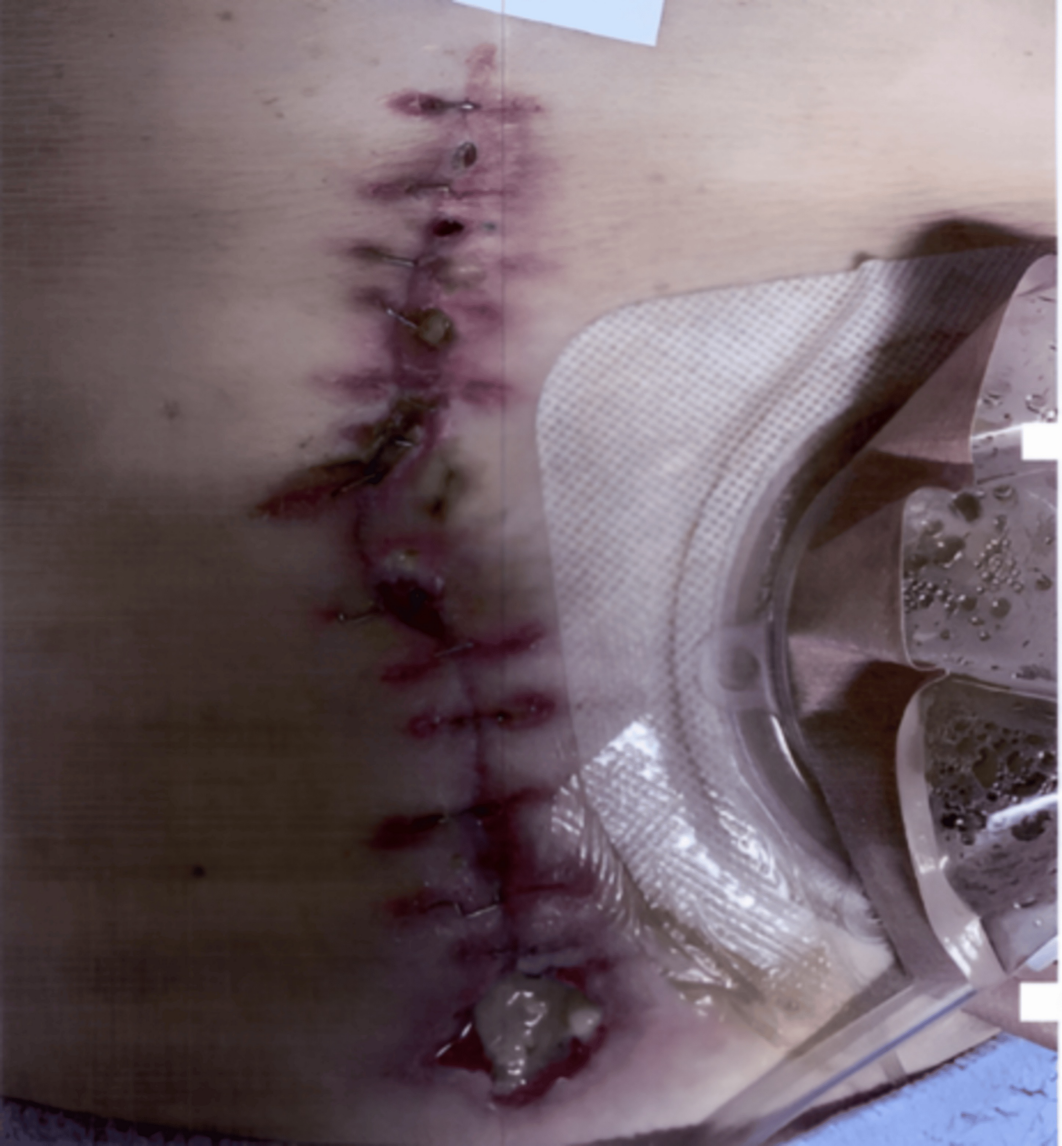
Post-operative wound check The clinical photograph obtained demonstrated a midline incision with staples in situ, with peri-incisional erythema, superficial dehiscence, and seropurulent drainage. A mucous fistula is present at the inferior terminus with surrounding tissue breakdown and mucous effluent.

The mucous fistula effectively decompressed the distal obstructed segment, allowing drainage of retained mucofecal material. The clinical photograph obtained during postoperative wound evaluation demonstrating a vertical midline laparotomy incision with skin staples in situ (Figure 3). The incision is notable for peri-incisional erythema with multiple foci of superficial wound dehiscence. There is associated seropurulent drainage along the incision, suggestive of superficial surgical site infection. At the inferior terminus of the incision, a mucous fistula is present, characterized by mucous effluent and surrounding macerated tissue with localized breakdown of the skin and subcutaneous layers. Early granulation tissue is visible within areas of separation. An ostomy appliance is positioned lateral to the incision.

Given the presence of persistent mucofecal drainage, the development of a surgical site infection was likely unavoidable. The mucous fistula, by nature, represents a chronic source of mucus production from the retained bowel stump, creating a continuously moist and contaminated environment that predisposes to bacterial colonization and subsequent infection. In this setting, the patient was empirically started on intravenous piperacillin-tazobactam, per infectious disease recommendations, after preliminary cultures demonstrated gram-negative organisms, with the goal of ensuring broad-spectrum coverage, including anaerobes. Blood and wound cultures with susceptibilities were obtained. On the following day, wound cultures grew Escherichia coli, prompting de-escalation to intravenous ampicillin/sulbactam based on sensitivity results. No growth was observed on blood cultures. Notably, the patient remained afebrile without leukocytosis throughout the course, exhibiting only localized incisional pain.

**Figure 4:**
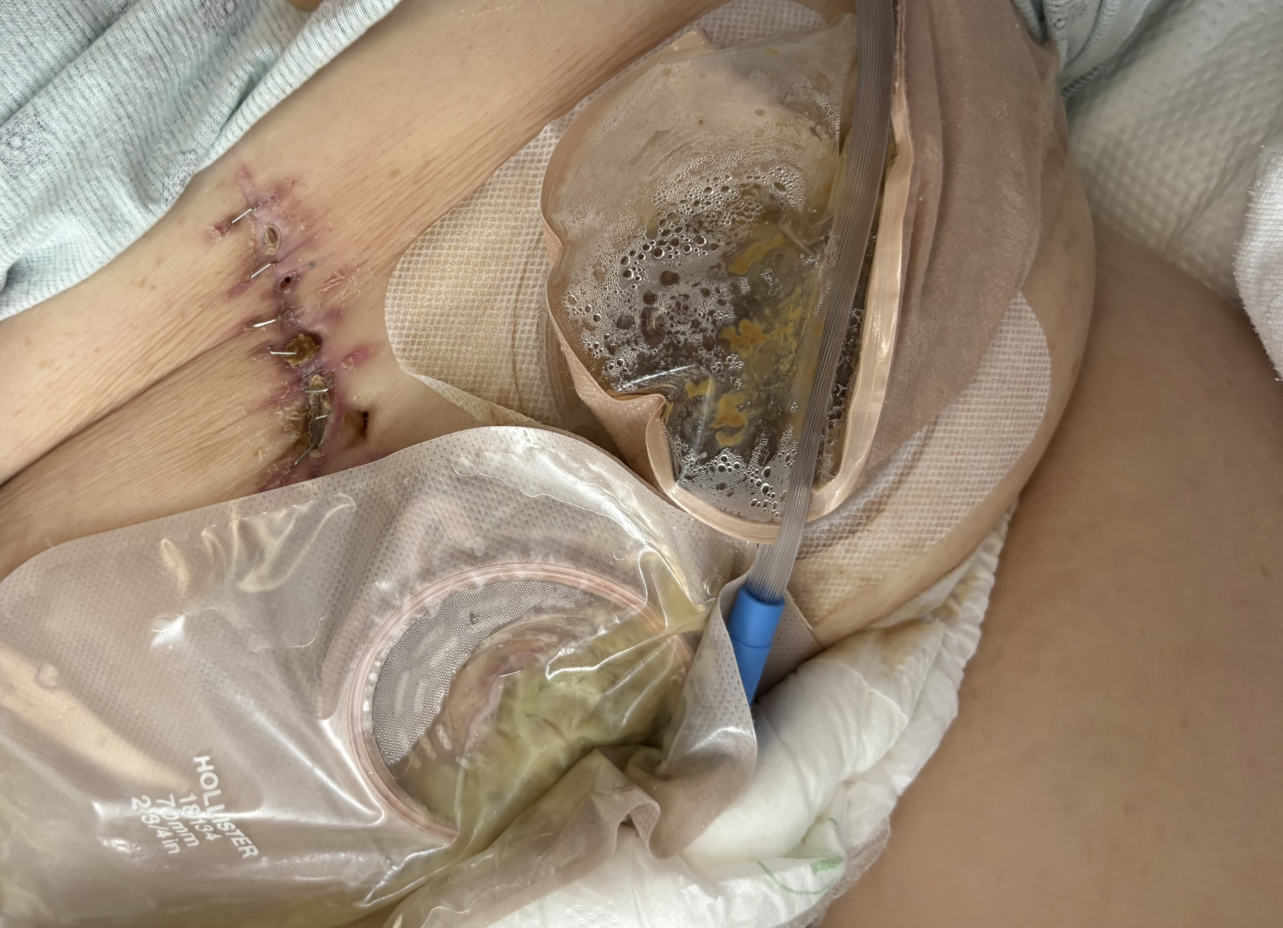
Postoperative midline incision with mucous fistula and colostomy at discharge Healing midline laparotomy incision with staples in situ. The superior aspect of the incision demonstrates primary intention healing with well-approximated edges. The mid-to-inferior section exhibits partial superficial dehiscence with visible fibrinous slough and serosanguinous exudate, transitioning from the inflammatory to the proliferative phase of wound healing. A mucous fistula along the incision drains mucopurulent material into an ostomy appliance, with an adjacent functioning colostomy and appropriate feculent output.

Prior to discharge, a clinical photograph of the surgical site was obtained, which showed improving peri-incisional erythema with areas of superficial separation and fibrinous slough, consistent with a resolving surgical site infection and a transition toward active wound healing (Figure 4).

The patient was ultimately discharged two weeks postoperatively on oral amoxicillin/clavulanate for a 14-day course. The midline laparotomy incision demonstrated appropriate healing, and the ostomy sites showed no evidence of peristomal erythema, induration, or infectious changes. The patient was subsequently discharged into the care of her son and returned to her hometown to continue her previously established oncologic follow-up, with plans for ongoing surgical evaluation of the mucous fistula and consideration of definitive management.

## Discussion

This case illustrates several important considerations in the management of advanced rectal cancer. First, it underscores the potential consequences of delayed definitive surgical treatment due to patient preference, emphasizing the importance of shared decision-making and thorough counseling regarding disease progression and emergency risks. Although patient autonomy must be respected, early and clear communication regarding the natural history of untreated obstructing rectal cancer may help prevent life-threatening presentations.

The primary surgical objective was a Hartmann’s procedure to resect the fungating rectal mass and alleviate the obstruction in this emergency presentation. Hartmann’s procedure is commonly employed in cases of obstructing or perforated colorectal disease, often based on intraoperative assessment of patient stability, anatomic constraints, and oncologic considerations [4]. However, intraoperative exploration via a midline incision revealed dense fixation of the mass to the pelvic sidewalls and sacrum. Notably, the choice of a midline incision was essential in this context, as it provided the necessary exposure to confirm the unresectability of the mass and allowed for the safe exteriorization of both the proximal and distal bowel limbs. Initial lysis of adhesions was performed specifically to assess the feasibility of establishing a safe dissection plane for resection; however, the degree of fixation rendered the mass unresectable without a high risk of catastrophic pelvic hemorrhage or visceral injury. Consequently, a formal Hartmann’s procedure was not pursued, as the immobility of the mass and the significant friability of the distal tissues rendered both a safe resection and a secure stump closure technically unfeasible, as it would have likely resulted in stump perforation and pelvic sepsis. While a diverting loop colostomy was an alternative, this procedure was anatomically precluded by the absolute lack of mobility in the distal segment. The fungating rectal mass was firmly incarcerated within the pelvic cavity, creating significant tension that prevented the rectal portion from being pulled through to the abdominal surface tension-free. Any attempt to force a loop under such tension would have risked ischemia or stomal retraction. By dividing the bowel, the proximal end was easily exteriorized as an end colostomy. The distal possessed just enough mobility to be brought to the surface as a mucous fistula at the inferior aspect of the incision. This approach prevented ischemia or stomal retraction and the accumulation of pressurized mucus within the compromised distal segment, thereby avoiding a "blowout" of the weakened rectal tissues.

This case highlights mucous fistula formation as a useful and under-recognized surgical strategy in adult colorectal emergencies. While mucous fistula formation is a well-established component of the surgical repertoire in pediatric surgery and for the management of inflammatory bowel disease [5], its application in the context of emergency adult oncologic obstruction represents a significant departure from standard practice. In most adult colorectal emergencies, the Hartmann’s procedure with a closed rectal stump remains the default; however, this case demonstrates a rare clinical scenario where unresectable pelvic fixation and distal tissue friability coexist. In such instances, the mucous fistula is utilized not as a routine staged maneuver but as a critical measure to decompress a fixed malignant segment that is otherwise inaccessible for resection or safe closure.

Current literature on mucous fistulas in adults is sparse, primarily consisting of isolated case reports rather than large-scale series or standardized protocols. Reported cases typically involve specific clinical scenarios, such as management of postoperative megarectum following congenital anorectal malformations or as part of staged management for fulminant colitis [6,7].

Limitations of this report include its single-patient design and the relative rarity of mucous fistula use in adult colorectal surgery, which limits generalizability. Long-term oncologic outcomes were not available, as definitive cancer management occurred at an outside institution.

## Conclusions

We present a rare case of septic large bowel obstruction due to advanced rectal cancer managed successfully with emergent colostomy and mucous fistula formation. When standard Hartmann’s procedure or diverting loop colostomy is not feasible, end colostomy with mucous fistula creation provides a safe and effective means of decompressing the excluded bowel and preventing catastrophic complications. This case reinforces the need for individualized surgical planning and early patient-centered counseling in rectal cancer management.
